# Educational approaches for patients with heart surgery: a systematic review of main features and effects

**DOI:** 10.1186/s12872-022-02728-0

**Published:** 2022-06-27

**Authors:** Leila Shahmoradi, Nafiseh Rezaei, Sorayya Rezayi, Mitra Zolfaghari, Babak Manafi

**Affiliations:** 1 Health Information Management Department and Medical Informatics, School of Allied Medical Sciences, Tehran University of Medical Sciences, Tehran, Iran; 2 Medical Library and Information Science, Tehran University of Medical Sciences, Tehran, Iran; 3 Department of Medical Library and Information Science, School of Allied Medical Sciences, Hamadan University of Medical Sciences, Hamadan, Iran; 4 Department of eLearning in Medical Education, Virtual School of Tehran University of Medical Sciences, Naderi Street, Keshavarz Blvd, Tehran, Iran; 5 Department of Heart Surgery, School of Medicine, Hamadan University of Medical Sciences, Hamadan, Iran

**Keywords:** Education, Heart surgery, PRISMA, Technology, Educational solutions

## Abstract

**Introduction:**

Patients who undergo heart surgery are exposed to mental and physical difficulties after discharge from hospital. They often need support and follow-up after discharge. The use of educational approaches or solutions before or after heart surgery can increase patients' knowledge on the post-operative complications and self-care. The main purpose of this systematic review is to determine the applications of educational approaches and investigate the effects of these approaches on patients with heart surgery.

**Method and materiel:**

A thorough search was conducted in Medline (through PubMed), Scopus, ISI web of science to select related articles published between 2011 and May 2022. All of the retrieved papers were screened according to the Preferred Reporting Items for Systematic Reviews and Meta-Analyses (PRISMA) checklist.

**Results:**

A total of 29 articles were obtained from the search, which included in this systematic review after being assessed based on inclusion and exclusion criteria. Most of the articles (*n* = 10, 34.48%) had been conducted in Canada and Iran, with the most significant number published in 2016. Out of 29 studies, 23 were experimental studies, and six were observational-analytical studies. The number of participants in the studies ranged from 11 to 600 (IQR1: 57.5, median: 88, IQR3: 190). In 28 (96.55%) studies, the educational approaches had a significant effect on clinical, economic or patient-reported outcomes. The greatest effect reported by the studies was related to clinical outcomes such as patient care improvement or change in clinical practice. The most effects in the patient-reported outcomes were related to improving patient satisfaction and patient knowledge. In terms of global rating scores, 17.24% of the included studies were considered as weak, 20.68% as moderate, and 62.06% as strong.

**Conclusion:**

The results of systematic review showed that the use of educational approaches by patients before and after heart surgery can have significant effects on reducing stress and financial burden, and increasing the quality of care and level of knowledge in patients.

## Introduction

Cardiovascular surgery, also called cardiac surgery or heart surgery, represents any surgical procedure that involves heart or blood vessels that carry blood to and from the heart [1]. These procedures are common in people who have heart disease or had a heart attack, stroke, or blood clot, and also those who are at high risk for developing these problems [2]. There are many types of heart surgery. The National Heart, Lung, and Blood Institute outline the most common coronary surgical procedures, which include Coronary Artery Bypass Grafting (CABG), heart valve repair or replacement, insertion of a pacemaker or an Implantable Cardioverter-Defibrillator (ICD), maze surgery, aneurysm repair, heart transplant, and insertion of Ventricular Assist Device (VAD) or Total Artificial Heart (TAH) [3]. CABG, also called coronary artery bypass, coronary bypass, or bypass surgery, is the most common type of heart surgery, so that more than 300,000 people have successful bypass surgery in the United States each year [4]. The high prevalence of these surgeries has many economic and medical consequences in most countries [5]. However, over a quarter of all CABG and/or VR patients are readmitted to hospitals with postoperative complications during the first three months of recovery. A possible explanation for developing postoperative complications during the recovery period is poor self-care behavior of patients [6].

Patient education is a crucial health intervention to encourage self-care behavior, but it may often lack the required effectiveness [7]. Remarkably, the dose, type, and timing of educational intervention may not be optimal in promoting self-care behaviors, which results in the onset of complications and increased hospitalizations that reduce health-related quality of life [8, 9].

The treatment team prescribes various measures that patients should perform and follow before and after heart surgery. Therefore, these patients need a comprehensive and robust education system to provide them with accurate and practical knowledge on actions they ought to undertake [8–10]. Nevertheless, previous studies and expert opinions indicate that despite the establishment of patient education systems, patients often do not correctly participate in the treatment and care, and also do not perform the actions required for their recovery [11]. These and similar issues are among the many challenges in this area that indicate the potential for systemic failures, which need to be addressed [12]. Numerous studies in patient training have shown that education related to patient health increases satisfaction and reduces anxiety and length of hospital stay [13, 14]. Accordingly, a thorough study of current educational systems or the development of a comprehensive system for patients after heart surgery is something that may not receive much attention in medical centers, but the need for such system is evident for these patients [10, 15].

Educational approaches are widely applied in many countries for training patients with heart surgery, and some papers have shown its clinical benefits [10, 14, 16] and positive effects on the survival of patients. The format of patient education differs depending on the degree of standardization and individualization [17, 18]. Different approaches and technologies can be used to educate patients after heart surgery. Technologies such as video resources, virtual reality-based environments, and educational media such as CDs, DVDs, and others are widespread in today's society. In addition to these materials, patients can use other sources such as electronic booklets and brochures [9, 19]. Therefore, a comprehensive study is needed to determine the effectiveness and characteristics of educational approaches or technologies used for education of patients with heart surgery.

## Material and methods

### Research question

- Has the use of educational approaches or technologies been effective in training patients with heart surgery?

- What are the characteristics of interventions used to educate patients with heart surgery?

### Search strategy and study selection

In this study, we conducted a systematic literature review of educational approaches or technologies used for training patients with heart surgery, using the Preferred Reporting Items for Systematic Reviews and Meta-Analyses checklist (PRISMA) [20]. Major scientific databases, including Web of Science, Medline (through PubMed), and Scopus were searched systematically, using keywords such as “patient education” and “heart surgery”. Consequentially, related articles published between January 1, 2011 and 21 May, 2022, were retrieved. After removing duplicates, titles and abstracts of retrieved articles, the remaining articles were reviewed by three authors (SR, NR and LS) independently based on inclusion criteria. Several titles and abstracts were also reviewed randomly by LS. In the next stage, full-text screening was carried out. The full texts of related citations were also retrieved and reviewed by three authors based on inclusion and exclusion criteria. Through a full-text review, the final decision was made by LS and BM if there was a disagreement between the authors in regard to the selection of eligible studies. A combination of Medical Subject Headings (MeSH) keywords and terms were used in the search strategy (Table 1).

**Table 1:** Keywords and search strategy for PubMed database

Database	Search strategy
PubMed	( ("patient education" OR "Patient Education as Topic"[Mesh] OR "Patient Education Handout"[Publication Type] OR " Education of Patients"[tw] OR (("Educational Technology"[Mesh] OR "Educational Technology" OR "Educational Technologies" OR "Teaching Materials"[Mesh] OR "Teaching Materials" OR "Teaching Material") AND ("Patients"[Mesh] OR "Patient" OR "Patients"))) AND ("heart surgery" OR "cardiac surgery" OR "Cardiac Surgical Procedures"[Mesh] OR "heart surg*" OR "cardiac surg*" OR "Arterial Switch Operation"[tw] OR "Cardiac Valve Annuloplasty"[tw] OR "Cardiomyoplasty"[tw] OR "Coronary Artery Bypass"[tw] OR "Coronary Atherectomy"[tw] OR "Coronary Balloon Angioplasty"[tw] OR "Fontan Procedure"[tw] OR "Heart Bypass"[tw] OR "Heart Massage"[tw] OR "Heart Transplantation"[tw] OR "Heart Valve Prosthesis Implantation"[tw] OR "Heart–Lung Transplantation"[tw] OR "Induced Heart Arrest"[tw] OR "Maze Procedure"[tw] OR "Mitral Valve Annuloplasty"[tw] OR "Myocardial Revascularization"[tw] OR "Norwood Procedures"[tw] OR "Pericardial Window Techniques"[tw] OR "Pericardiectomy"[tw] OR "Pericardiocentesis"[tw] OR "Transcatheter Aortic Valve Replacement"[tw] OR "Transmyocardial Laser Revascularization"[tw] OR "Arterial Switch"[tw] OR "Cardiac Valve Annuloplast*"[tw] OR "Cardiomyoplast*"[tw] OR "Coronary Artery Bypass*"[tw] OR "Coronary Atherectom*"[tw] OR "Coronary Balloon Angioplast*"[tw] OR "Heart Bypass*"[tw] OR "Heart Transplant*"[tw] OR "Heart Valve Prosthesis Implant*"[tw] OR "Heart–Lung Transplant*"[tw] OR "Mitral Valve Annuloplast*"[tw] OR "Myocardial Revascular*"[tw] OR "Norwood Procedure"[tw] OR "Pericardial Window Technique"[tw] OR "Pericardiectom*"[tw] OR "Transcatheter Aortic Valve Replacement*"[tw] OR "Transmyocardial Laser Revascular*"[tw] OR "Heart/surgery"[mesh] OR "Heart Diseases/surgery"[mesh]) From 2011 to 2022

### Criteria used for the selection of articles

Studies with the following inclusion and exclusion criteria were included in this review.

#### Inclusion criteria

**Fig. 1:**
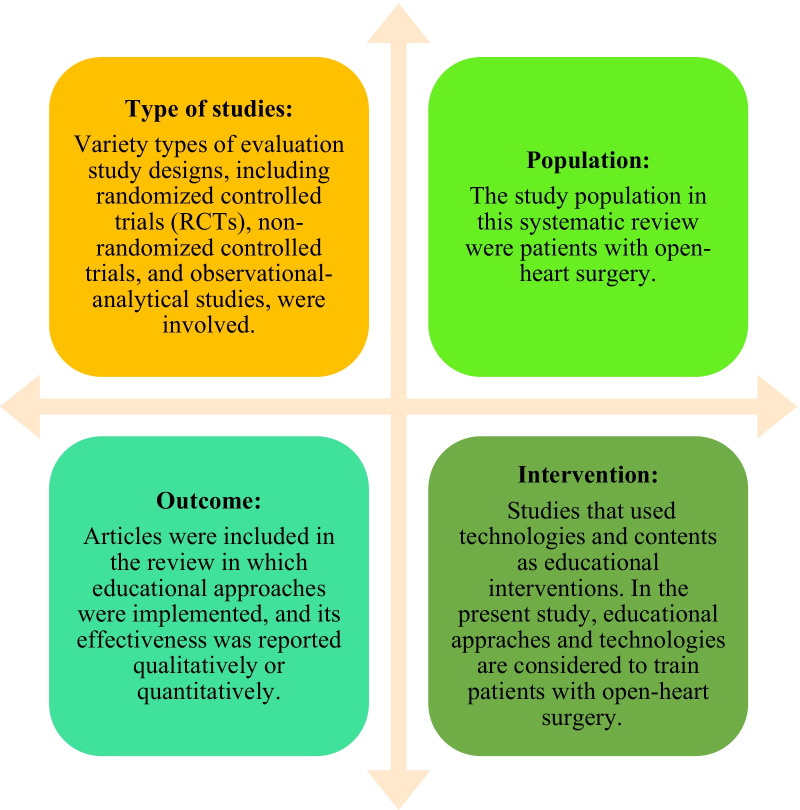
Inclusion criteria in this review

The inclusion criteria for the articles are presented in Fig. 1.

#### Exclusion criteria

Articles were excluded if they met the following criteria:

- Studies published in non- English language.
- Studies that were not original research (such as book chapters, letters to the editor, reviews or meta-analysis, short briefs, reports and commentaries).
- Studies that did not examine the impact of educational technologies or approaches on patients.
- Studies that their full text was not available.

### Data extraction

A form in Excel was designed to extract data from included articles. Some classifications were used to classify and analyze the included papers. This classification comprised of general information and specific details. General information included author’s names, publication date, country and journal’s name. Specific information included number of participants, mean age, gender, study participants, intervention group (I), comparison (C) group, study design, content of patient education and theory, applied intervention, effectiveness, main finding, and key outcome.

### Data analysis

In order to describe and compare the articles’ results, a narrative synthesis was applied, but meta-analysis was not done due to the diversity of outcomes. We classified outcomes into three main categories of clinical, economical, and patient-reported outcomes. The effect of educational materials on patients with heart surgery was summarized based on three categories: Positive without statistical argument, Positive (statistically significant), No effect (not statistically significant).

### Risk of bias and quality assessment

For risk of bias appraisal and quality assessment, we used The Effective Public Health Practice Project (EPHPP) tool to evaluate the quality of selected articles. This tool was preferred because of its ability to evaluate the quality of various quantitative studies related to public health issues or the use of technology in the health industry. In each study, the risk of bias was reckoned for six components; (1) selection bias; (2) study design; (3) confounders; (4) blinding; (5) data collection method; and (6) withdrawals and dropouts [21]. These six components were ranked as strong, moderate, and weak on the three-point Likert scale. Overall methodological quality is rated as weak (two or more poor ranking of individual scale), moderate (one weak individual scale rating), and strong (no weak scale rating). For bias and quality assessment, two authors (SR and NR) investigated each paper, and any disagreements were resolved by discussion with LS and MZ.

## Results

**Fig. 2:**
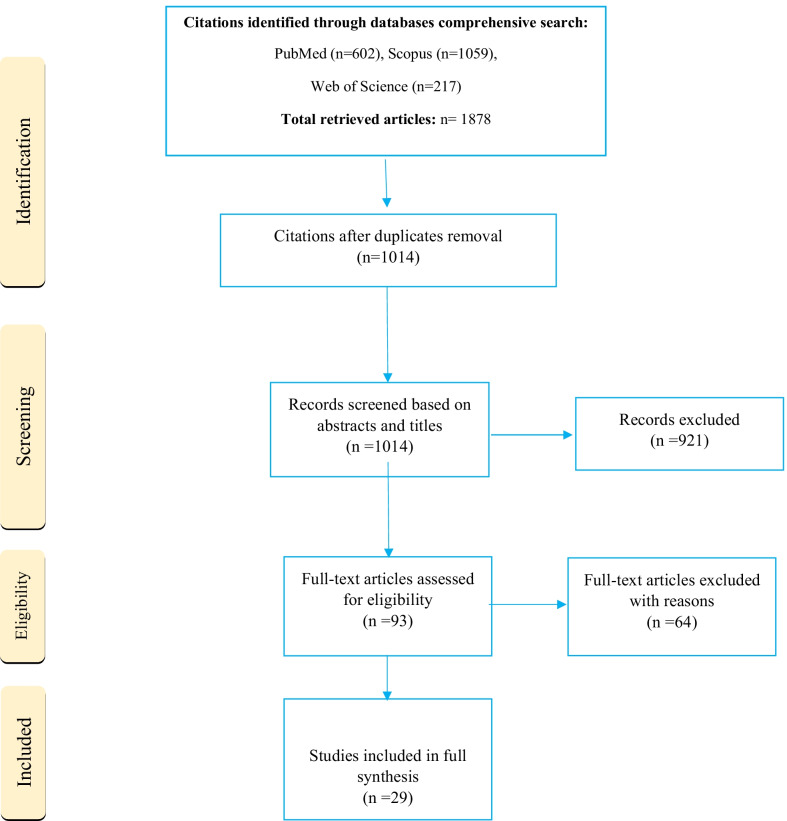
The PRISMA diagram for the records search and study selection

Earlier searches in various scientific databases yielded 1878 studies. After duplicate removal, 1014 citations were remained, from which 921 were omitted due to their irrelevancy in the abstract and titles screening stage. After reviewing the full-text of the related citations and applying the exclusion and inclusion criteria, 29 studies were included in this systematic review. The flow diagram related to the identification of eligible articles is shown in Fig. 2. It should be noted that a summary of the key results of papers is described in Table 2 based on the predefined classification elements.

**Table 2:** General characteristics of the included studies (*N* = 29)

#	Author	Country	Year	Number of participants, mean age, gender	Study participants	Study design and duration	Content of patient education and theory	Applied intervention	Main finding	Key effects	Outcome
1	Bjørnnes, A. K.[12]	Norway	2017	♦ 416 participants (23% women), M &F	♦ Patients > 18 years of age, able to read and write Norwegian and scheduled for elective coronary artery bypass surgery (CABG) and/or valve surgery were consecutively invited to participate in the study from March 2012 through to September 2013	Randomized controlled trial, From March 2012 to September 2013	Educational pain management booklet	Booklet and telephone	♦ The pain intensity did not decrease compared to the control	♦ Patient care improvement	• No statistically significant differences between the groups were observed in terms of the outcome measures following surgery
2	Cook, D. J.[22]	United States	2014	♦ 149 patients with a mean age of 68 years	♦ Utilized 5,267 of 6,295 (84%) Patients were provided with iPad® (Apple ®, Cupertino, CA) tablets that delivered educational modules as part of a daily "to do" list in a plan of care	Observational study without control	Educational modules as part of a daily "to do" list in a plan of care	Mobile application	♦ The combination of mobile computing with a content management system allows for dynamic, modular, personalized, and "just-in-time" education in a highly consumable format	♦ Patient care improvement	• Mobile phone or tablet can be effective in educating the patient
3	De Oliveira, A. P. A.[23]	Brazil	2016	♦ 90 patients, 45 in each group, mean age was 61.64 yrs. in CG and 63.87 yrs. in IG, most comprising male patients (68.9%)	♦ Patients undergoing myocardial revascularization (CABG) surgery	Randomized controlled trial, from May 2012 to August 2013	Bedside orientation	Video resources	♦ Orientation performed with the aid of video resources is more effective for knowledge retention in preoperative patients, compared to verbal orientation alone	♦ Patient knowledge improvement	• The use of video resources such as short films and slides can be effective in educating patients
4	Fredericks, S.[24]	Canada	2013	♦ 33 patients, the mean age was 66 ± 10 years (range:32–88 years)	♦ Study participants who had suffered a myocardial infarction, underwent revascularization, or who had angina pectoris or coronary heart disease. Individuals who underwent CABG or VR were underrepresented	A descriptive study, the first three months of recovery	Post-operative program	Telephone	♦ Increasing the number of times education is provided may reduce the number of hospital readmissions	♦ Reduction in hospital readmissions ♦ Reduction in cost of care	• Teaching the patient self-care after heart surgery reduces the likelihood of recurrence
5	Guo, P.[18]	United Kingdom	2012	♦ 153 adult patients, Mean (SD) age in years 52.3 (15.99) 52.0 (16.12)	♦ Elective cardiac surgery were eligible for the trial if they were able to speak, read, and write Chinese. Cardiac surgery included coronary artery bypass grafting, valve surgery, congenital and other open-heart surgery	Randomized controlled trial, From March 2012 to September 2013	Usual care plus an educational booklet at discharge with supportive telephone	Booklet and telephone	♦ This form of preoperative education is effective in reducing anxiety and depression among Chinese cardiac surgery patients	♦ Reduction of patients' depression and anxiety	• Preoperative education is effective in reducing patients' stress and anxiety
6	Hoseini, S.[8]	Iran	2013	♦ 70 patients, Mean and standard deviation of age in the intervention and control groups were 60. 86 ± 9 45 and 59 77 ± 7 29	♦ Undergoing CABG surgery in two hospitals in Shiraz. The patients were divided into two equal groups, the control and intervention	Randomized controlled trial, six weeks	Educational program after surgery	Audiotape	♦ The mean scores obtained in both anxiety and depression dimensions were significantly different between the intervention and control groups	♦ Patient satisfaction improvement	• Audio tape containing postoperative training is effective in-patient self-care
7	Kadda, O.[25]	Greece	2016	♦ 250 patients, Intervention Group (Age): Men (*n* = 184) Women (*n* = 65), (years): Men (64.2 yrs.) Women (70 yrs.) ♦ Control Group (Age): Men (n = 187) Women (*n* = 63), (years): Men (62.8yrs) Women (66.7 yrs.)	♦ Valvular heart disease from different causes, like endocarditis, rheumatic heart disease, or replacement with combined CABG)	Randomized, nonblind intervention, with 1-year follow-up	Specific educational information for postoperative rehabilitation	Booklet	♦ Lifestyle nursing intervention immediately after open heart surgery had a beneficial effect on men 1 year after the surgery but not on women	♦ Lifestyle changes	• Educating patients about postoperative care and lifestyle and heart rehabilitation by nurses can be effective in the healing process
8	Lai, V. K. W.[26]	Hong Kong	2021	♦ 100 (50 treatment, 50 control) patients and ♦98 (49 treatment, 49 control) family members, ♦94 (48 treatment, 46 control) patients ♦ 94 (47 treatment, 47 control) family members completed the trial	♦ Elective coronary artery bypass grafting valve surgery patients and their family members	Randomized controlled trial, From September 2015 to August 2017	Structured information in a preoperative video and ICU tour	Video resources	♦ Providing comprehensive preoperative information about ICU to elective cardiac surgical patients improved patient and family satisfaction levels and may decrease patients' anxiety levels	♦ Patient satisfaction improvement ♦ Reduction of patients' depression and anxiety	• Preoperative education of the patient and the patient's family can be effective in the patient's recovery process and reduce anxiety
9	Lai, V.K.W.[27]	Hong Kong	2016	♦ 100 patients (50 patients in each group)	♦ Patients undergoing elective cardiac surgery on patient and family satisfaction with care and decision-making in the ICU	Randomized controlled trial, 30 days	A preoperative patient education intervention	Video resources	♦ Preliminary results indicated that patients and their families were satisfied with the training on care, and secondary results indicated a reduction in anxiety	♦ Patient satisfaction improvement ♦ Reduction of patients' depression and anxiety	• Preoperative education for patients and their families will improve the performance of postoperative care and reduce their anxiety
10	Lowres, N.[28]	Australia	2016	♦ 42 participants (mean age 69 ± 9 years, 80% male)	♦ They had no prior history of atrial fibrillation (AF) and were discharged home in stable sinus rhythm	Cross sectional study, March 2014 and July 2015	5- to 10-min practice session was required to successfully learn to use the iECG	Educational module on handheld portable ECGs	♦ Using this technology can help reduce the recurrence of the disease and control stress to the patient	♦ Reduction of patients' depression and anxiety	• New technologies in the field of self-care for patients help Anna and their families in controlling illness and stress
11	Martorella, G.[29]	Canada	2013	♦ 30 patients, 20% of women and 80% of men with a mean age of 65 years	♦ Adults undergoing cardiac surgery, to promote the self-management of postoperative pain	Observational study without control, over 4 months in 2010	The development and validation of a tailored Web-based intervention for postoperative pain self-management in adults who underwent cardiac surgery	Virtual environment	♦ Patient empowerment is complementary yet crucial in the current context of care and may contribute to improved pain relief	♦ Patient care improvement	• The use of new information technologies can personalize patient care and provide more complete patient care
12	McGillion, M.[30]	Canada	2020	♦ 11 patients, all patients were over 65 years of age, the majority of patients were male	♦ Patients recovering from cardiac or major vascular surgery	Randomized controlled trial, 30 days	Educational program after surgery	Virtual environment	♦ The need for additional opportunities to practice in order to become comfortable and proficient in the use of these systems	♦ Clinical practice Change	• The use of new remote care technologies will create personalized and continuous care
13	Melholt, C.[31]	Denmark	2018	♦ 49 cardiac patients, mean age of 60.64 ± 10.75 years, and 82% of the respondents were males	♦ Cardiac patients	Observational study without control, September 2014 and February 2015	Not mentioned	The interactive ‘Active Heart’ web portal	♦ The patients’ eHealth literacy skills increased during the trial period	♦ Patient care improvement	• Distance and online education can be effective in increasing self-care and rehabilitation skills after heart surgery
14	Moghimian, M.[32]	Iran	2019	♦ 80 patients, age range between 40 and 70 years	♦ Being a candidate for coronary artery bypass graft, first time open-heart surgery, lack of cognitive problems such as dementia, lack of physical disability such as blindness or deafness, age range between 40 and 70 years	Before-after Design, study, in 2017	Digital storytelling on the anxiety	Digital storytelling media	♦ Storytelling in multimedia environments can reduce the tension experienced by many presurgical patients	♦ Reduction of patients' depression and anxiety	• The use of multimedia tools is effective in educating patients and reducing their anxiety
15	O'Brien, L.[33]	Australia	2013	♦ 375 people who had undergone cardiac surgery, (70.1%) were men and 112 (29.9%) were women, mean age 66 yrs	♦ Patients who underwent elective cardiac surgery	Cross sectional study, in 2009–2010	Both pre-operative written information and post-operative education relating to post-operative precautions	Booklet	♦ Multidisciplinary written pre-surgery education appears to be providing patients with a good understanding of what to expect following surgery	♦ Patient care improvement ♦ Reduction of patients' depression and anxiety ♦ Reduction in cost of follow up	• Providing education to patients before surgery and being aware of their expectations can lead to faster recovery and reduced anxiety
16	Pazar, B.[34]	Turkey	2020	♦ 200 patients, 100 intervention group (77% male) ♦ Control group100 (72% male)	♦ Preoperative education of cardiac patients on hemodynamic parameters, comfort, anxiety and patient-ventilator synchrony	Randomized controlled trial, from June 2015 to April 2016	Preoperative education on mechanical ventilation	Brochure	♦ The participants in the intervention group who received education had higher patient-ventilator synchrony, comfort and hemodynamic stability levels, anxiety levels when they were under mechanical ventilation, showing that results were better in the intervention group than the control group	♦ Patient care improvement ♦ Reduction of patients' depression and anxiety	• Educating patients about ventilator use and postoperative care can reduce postoperative complications and reduce patients' anxiety
17	Salehmoghaddam, A.[5]	Iran	2016	♦ 60 patients, means age of patients were 57.5 and 56.2 yrs. in the intervention and the control groups	♦ All patients undergoing open heart surgery hospitalized at open heart surgery	Randomized control trial, from September 2015 to December 2015	Instructional videos on respiratory function	Video resources	♦ The instructional videos rather than pamphlet and face-to-face training to improve postoperative respiratory function in patients undergoing open heart surgery	♦ Patient knowledge improvement ♦ Patient care improvement	• The use of educational videos is more attractive than face-to-face training and written materials. It is also possible to prepare different videos according to the level of education of individuals
18	Wakefield, B.[35]	United states	2014	♦ Intervention (*N* = 43), Usual-care (*N* = 12), Mean age (yrs.) 63.7, 63.8	♦ Remote cardiac rehabilitation participants (*n* = 48) received education and assessment during 12 weeklies by telephone calls. Data were compared with those for face-to-face CR program participants	Non-randomized control trial, From August 2010 through August 2011	Remote cardiac rehabilitation content	Telephone	♦ Remote CR is a viable alternative to bring services closer to the patient	♦ Hospitalization reduction	• Providing telephony-based services can reduce the need for ongoing patient care and help those who are unable to attend hospital
19	Pakrad, F. [36]	Iran	2021	♦ Intervention group (*N* = 44) control group (*N* = 44), Age (62.6 ± 8.1,62.9 ± 9.8), Sex (Male36, Female 8), (Male38, Female6)	♦ Patients Who Have Undergone Coronary Artery Bypass Surgery	Randomized controlled trial, from October 2019 to April 2020	An educational booklet regarding risk factor management was provided to these participants	Booklet and telephone	♦ The CCM was effective in not only improving the primary and secondary outcomes in this trial, but also affecting the process indicators as hypothesized. Indeed, CR participants exposed to the CCM had significantly more positive perceptions of the quality of their care and its continuity	♦ Patient care improvement. ♦ Reduction of patients' Depression, Anxiety and Stress	• This trial demonstrates that applying the CCM to CR in a hybrid delivery model results in clinically significant improvements in QOL and functional capacity, as well as reduced rates of rehospitalization
20	Mayer-Berger, W. [37]	Germany	2014	♦ Intervention group (*N* = 271) control group (*N* = 329), (gender): Men (*n* = 246), Mean age (yrs.) 49.2 ± 5.7, 49.1 ± 5.4	♦ Coronary artery disease (CAD) patients of low educational level compared to usual care after surgery	Randomized controlled trial, 5 years to 31 December 2010,	The contents of the educational program were selected based on literature, especially the manuals My Heart, My Life (National Heart Foundation of Australia2008) and The Heart Manual (Lothian Health Board2007) about the risk factors for CAD and correct use of medication	Telephone	♦ Patients in the IG showed better 3-year risk profile outcomes, the PROCAM score increased by 3.0 (IG)	♦ Patient care improvement	• This long-term secondary prevention program with inpatient rehabilitation at the beginning and telephone reminders for a 3-year period was successful. There were significant differences in health-related quality of life
21	Furuya, R. K. [17]	Brazil	2014	♦ Intervention group (*N* = 30) control group (*N* = 30), Age (63.3, 60.6), Sex (Male 60.0, Female40.0), (Male 53.3, Female 46.7)	♦ Sixty patients who were preparing for their first percutaneous coronary	Quasi-experimental, between August 2011–June 2012	Booklet 1: ‘Percutaneous Transluminal Coronary Angioplasty’ Booklet 2: ‘Going home after your coronary angioplasty’ Booklet 3: ‘How to take care of your heart and your health’	Booklet and telephone	♦ The educational program with telephone follow-up is potentially an effective strategy to provide motivation and emotional and social support to the patient, leading to reduced anxiety symptoms with trends toward improvement in some domains of health status	♦ Patient care improvement	• The educational program with telephone follow-up is a promising
22	Kalogianni, A. [38]	Greece	2015	♦ Intervention group(*N* = 205) control group (*N* = 190), Age (65.9, 65.1), Sex (Male 145, Female 60), (Male 140, Female 50)	♦ All patients admitted for elective cardiac surgery included CABG, valve replacement, ascending aortic aneurysm repair or a combination of these	Randomized controlled, from May 2011 until January 2014	It included information about anatomy, function, and surgical diseases of the heart, the open-heart surgery, the hospital, the perioperative period and process and emphasized the self-care of patients	Booklet	♦ The state of anxiety on the day before surgery decreased only in the intervention group	♦ Patient care improvement ♦ Reducing readmissions or length of stay ♦ Reduction of patients' Depression, Anxiety and Stress	• Preoperative education delivered by nurses reduced anxiety and postoperative complications of patients undergoing cardiac surgery, but it was not effective in reducing readmissions or length of stay
23	Wang, L. W. [39]	Taiwan	2016	♦ Intervention group(*N* = 20) control group (*N* = 40), Age (61.32 ± 13.4), Sex (men (66.7%)	♦ The patients underwent first-timeCABGor heart valve surgery	Quasi-experimental study, from August to December 2010	The intervention comprised exercise training with multimedia DVDs and printed booklets distributed by the researchers	Multimedia DVDs and printed booklets	♦ These results suggest that our multimedia exercise training program has clinical benefits on physical activity and fitness for patients admitted to phase 1 exercise training after first-time, uncomplicated cardiac surgery	♦ Patient care improvement	• Multimedia exercise training program safely improved distance walked in the 6MWT, heart rate recovery, and self-efficacy at hospital discharge in patients after heart surgery and maintained them improvement in 6MWT and self-efficacy 1 month later
24	Bikmoradi, A. [40]	Iran	2017	♦ Intervention group (N = 36) control group (*N* = 35), Age (62 ± 7.41, 64.03 ± 7.77), Sex (Male 27, Female 9), (Male22, Female13)	♦ Patients who had undergone CABG	Quasi-experimental study, in 2014	Included an emphasis on education and counseling about the correct administration of medications, recommendations on diet and physical activity levels, avoiding heart disease risk factors and smoking, pain management, care of the surgical incision, maintenance of balanced mental health, and maintenance of balanced bowel movements, sleep and vital signs	Telephone	♦ There was significant and positive deference between the two groups in favor of the telephone counseling after the intervention	♦ Patient care improvement ♦ Patient satisfaction improvement	•Telephone counseling could be a cost-effective patient counseling plan for therapeutic adherence after coronary artery bypass surgery in order to improve the patients’ quality of life
25	Widmer, R. J. [15]	USA	2017	♦ Intervention group (*N* = 37) control group (*N* = 34), Age (62.5 ± 10.7, 63.6 ± 10.9), Sex (Male29), (Male29)	♦ Patients entering three months of Mayo Clinic CR after heart surgeries randomized in a 1:1 fashion via computer generated sequence to standard CR versus standard CR + DHI	Randomized clinical trial, from august 2013 and February 2015	Cardiac rehabilitation platform asking the patients to report of dietary and exercise habits throughout CR as well as educational information toward patients' healthy lifestyles	Mobile application	♦ CR platform asking the patients to report of dietary and exercise habits throughout CR as well as educational information toward patients' healthy lifestyles	♦ Patient care improvement ♦ Patient knowledge improvement	• The study suggests a role for DHI as an adjunct to CR to improve secondary prevention of CV disease
26	Coskun, H. [41]	Turkey	2016	♦ Intervention group (*N* = 90) control group (*N* = 90), Age (66 ± 3, 50 ± 9), Sex (Male 64, Female 26), (Male60, Female30)	♦ Patients who underwent cardiovascular surgery	Randomized clinical trial, from November 2011 and June 2012	Discharge education	Booklet	♦ Both written and verbal discharge training increased the knowledge levels	♦ Patient care improvement ♦ Hospitalization reduction ♦ eduction in cost of care	• To solve the problems after discharge, which may reduce the number of patients presenting at hospital and in turn, related healthcare costs
27	Ramesh C. [21]	India	2020	♦ Intervention group (*N* = 65) control group (*N* = 65), Age (57.62, 57.46), Sex (Male55, Female 10), (Male53, Female 12)	♦ Patients with CABG surgery	Randomized controlled trial, six months in 2018	The health promotion model (HPM) seeks to enhance one’s health and well-being. The HPM concentrates on the following three areas: (i) Individual characteristics and experiences, (ii) behavior-specific cognitions, and (iii) behavioral outcomes	Video resources	♦ Nurses should use well-structured content to teach patients before CABG surgery and spend enough time on patient education regular	♦ Reduction of patients' Depression, Anxiety and Stress ♦ Patient care improvement	• Patient education effectively decreases anxiety, pain, and fatigue and improves self-efficacy and quality of life in patients undergoing CABG surgery
28	Fahimi, K. [42]	Iran	2018	♦ Intervention group (*N* = 55) control group (N = 55), Age (57.84 ± 13.117, 57.69 ± 11.23), Sex (Male28, Female 27), (Male28, Female 27)	♦ The inclusion criteria were experiencing the coronary artery bypass graft for the first time and non-development of postoperative cardiogenic shock or myocardial rupture	Randomized clinical trial, in 2016	Multimedia education on postoperative delirium in patients undergoing a coronary artery bypass graft	Booklet/The mobile application	♦ The results indicated that the highest incidence of delirium was observed on the first day after surgery in the intervention group (7.3%) and on the morning of the second day after surgery (14.5%) in the control group	♦ Reduction of patients' post-operative delirium	• Considering the lower incidence of post-operative delirium in patients who experienced multimedia education rather than control group, the use of this non-pharmaceutical method is recommended to prevent delirium in such patients
29	Fredericks, S. [43]	Canada	2013	♦ Experimental group (*N* = 17) control group (*N* = 17) Mean age (yrs.) 66.2, 65.6 ± 8.3; men (77.8%)	♦ CABG and/or VR surgery for the first time, spoke English; To had access to a working phone following hospital discharge	Randomized controlled trial, 3 months	The topic areas identified on the PLNS (Patient Learning Needs Scale) are reflective of CABG and VR patients’ learning needs	Telephone	Educational patients to reduce the number of hospital readmissions and complications at three months following hospital discharge	♦ Patient care improvement ♦ Reducing readmissions or length of stay	An impact on reducing hospital readmission rates and complications during the initial home recovery period

Coronary Artery Bypass Grafting (CABG), Control Group (CG), Interventional Group (IG), Standard Deviation (SD), Cardiac Rehabilitation (CR), Cardio Vascular Disease (CAD), Quality of Life (QoL), Atrial Fibrillation (AF), Continuous Care Model (CCM), Intensive Care Unit (ICU), Valve Replacement (VR), Electrocardiogram (ECG)

### Study characteristics

**Fig. 3:**
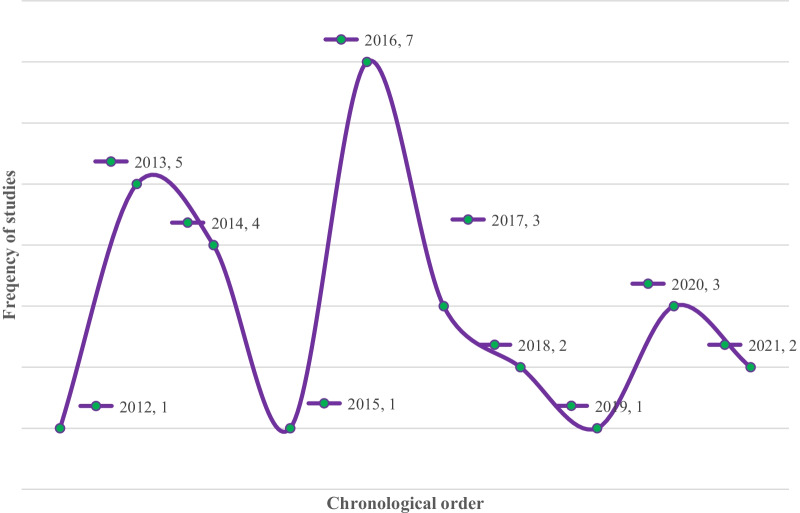
The distribution of papers by publications year

All selected papers that met the inclusion and exclusion criteria had been published in 27 reputable journals. All the names of journals are listed in Table 3, based on their frequency and quartile. Notably, 23 (79.31%) included investigations were published in top quartile one journals. It should be noted that the oldest and newest papers had been published in 2013 and 2021, respectively. The distribution of papers based on publication year is depicted in Fig. 3. As seen in the figure, the largest number of articles (*n* = 7, 24.13%) had been published in 2016.

**Table 3:** Distribution of journals by quartile and frequencies

Journal name	Column Labels				
Row labels	Q1	Q2	Q3	Without Q	Grand Total
American heart journal	1				1
Angiology	1				1
Archives of physical medicine and rehabilitation	1				1
Australian occupational therapy journal	1				1
BMJ open	1				1
BMJ quality & safety	1				1
CIN: Computers, informatics, nursing		1			1
Complementary therapies in clinical practice		1			1
European journal of cardio-thoracic surgery	1				1
European journal of cardiovascular nursing	2				2
European journal of preventive cardiology	1				1
Intensive and critical care nursing	1				1
International journal of health promotion and education			1		1
International journal of nursing studies	1				1
Journal of advanced nursing	1				1
Journal of cardiovascular nursing	1				1
Journal of clinical nursing	1				1
Journal of evidence-based care			1		1
Journal of medical internet research	1				1
Journal of the brazilian medical association				1	1
Nursing in critical care	1				1
Patient education and counseling	2				2
Rehabilitation nursing		1			1
Telemedicine and e-health	1				1
Telemedicine journal and e-health	1				1
The journal of cardiovascular nursing	1				1
Western journal of nursing research	1				1
Grand total	23	3	2	1	29

### The distribution of articles based on the countries

**Fig. 4:**
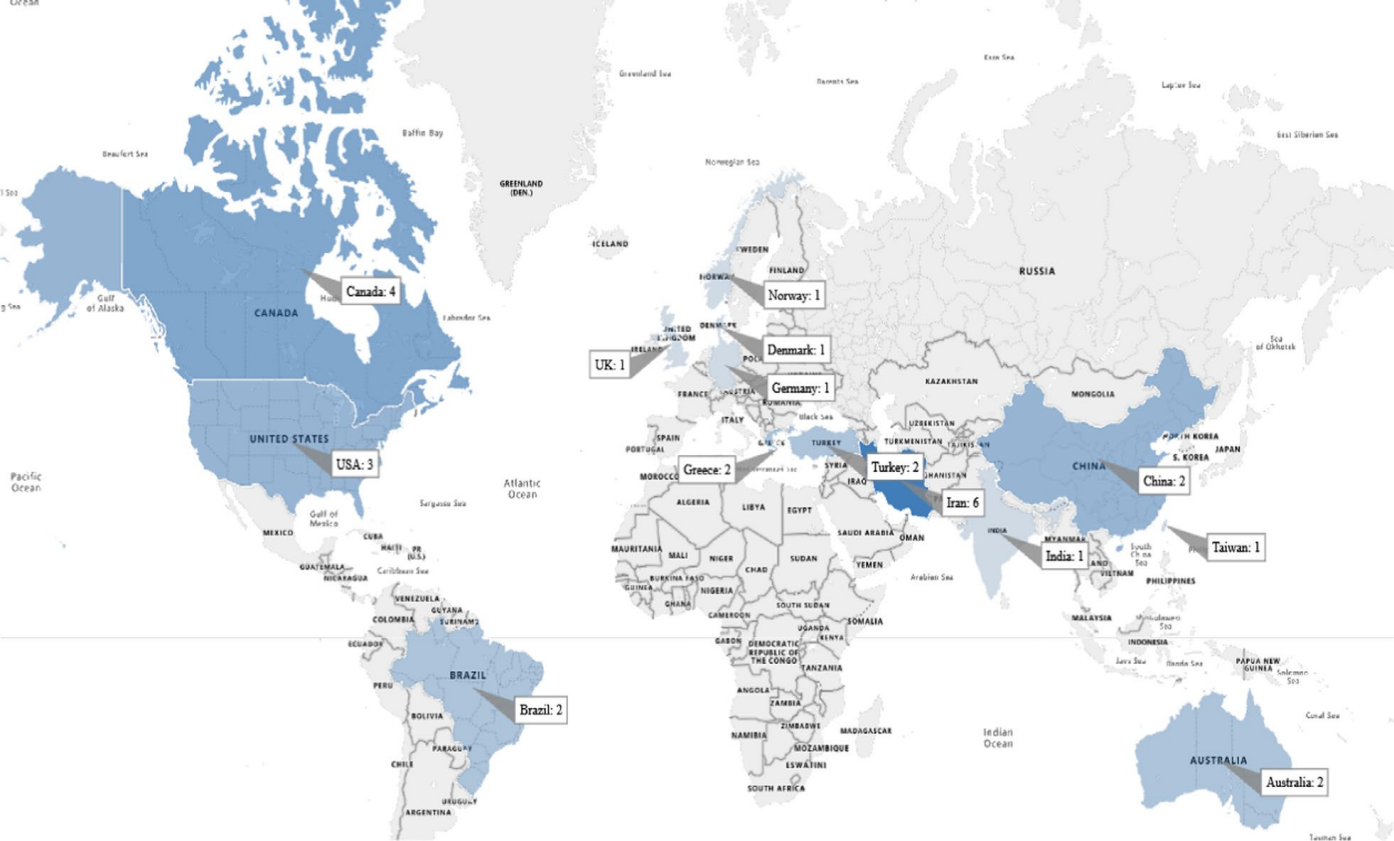
The distribution of papers by countries

The selected papers had been published in 14 countries. The distribution of studies based on country is shown in Fig. 4, based on the worldwide map. As it turns out, Iran and Canada had the highest frequency (*n* = 10, 34.48%) compared to other countries.

### Distribution of papers based on sample size and type of studies

The number of participants in the studies ranged from 11 to 600 (IQR1: 57.5, median: 88, IQR3: 190). It should be noted that the study design was mostly experimental in the form of Randomized Controlled Trial (RCT), (*n* = 18, 62.06%). The distribution of studies based on the study design and type is shown in Table 4.

**Table 4:** Distribution of papers based on study design

Row Labels	Frequency
**Experimental**	**23**
Before-after Design	1
Quasi-experimental	3
Non-randomized controlled trial	1
Randomized controlled trial	18
**Observational-analytical**	**6**
Cross sectional study	2
Observational study without control	4
**Total**	**29**

### Distribution of papers based on type of applied materials

**Fig. 5:**
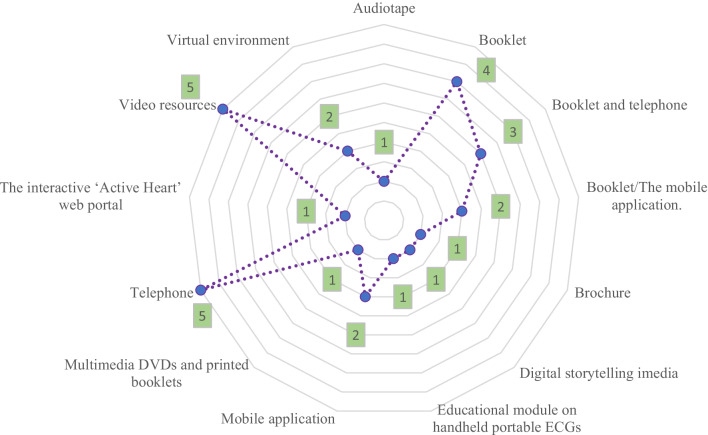
Distribution of articles based on the type of used solutions

In the selected studies, different technologies and educational contexts had been used. The distribution of articles based on the type of applied approaches is shown in Fig. 5. As can be seen in the figure, booklet (electronic booklets), telephone and video resources had been the most widely used educational resources.

### Effectiveness of educational interventions

The effectiveness of educational interventions on patients with heart surgery was summarized based on three categories:

- No effect (not statistically significant)
- Positive without statistical argument
- Positive with statistically significant

As can be seen, in the 22 (75.86%) studies, the employed educational interventions had a statistically significant effect on key outcomes such as increasing the level of knowledge of patients, reducing the length of hospital stay, increasing the level of satisfaction of patients and their families, and so on. Notably, in six examinations (20.68%), the applied educational approaches had positive effects on above-mentioned measures without statistical argument. In one study, the educational platform provided to patients did not affect the quality of care and patients' level of knowledge. Table 5 lists the key factors along with their effectiveness.

**Table 5:** Effects of educational interventions on key factors

Outcome category	Outcomes	Effects		
		Positive effect (without statistical argument)	Positive effect (statistically significant)	References
**Clinical outcomes**	Clinical practice change	1		[30]
	Hospitalization reduction		2	[35, 38]
	Reduction of patients' depression and anxiety	1	9	[18, 21, 26–28, 32–34, 36]
	Reduction of patients' post-operative delirium		2	[36, 42]
	Patient care improvement	2	15	[5, 12, 15, 17, 21, 22, 29, 31, 33, 34, 36–41, 43]
**Patient-reported outcomes**	Patient satisfaction improvement	1	4	[8, 26, 27, 36, 40]
	Family satisfaction improvement		2	[26, 36]
	Patient knowledge improvement	1	2	[5, 15, 23]
	Lifestyle changes		1	[25]
**Economic outcomes**	Reduction in cost of care		3	[24, 36, 41]
	Reduction in hospital readmissions and stays		5	[24, 36, 38, 41, 43]
	Reduction in cost of follow up		1	[33]
**Total effects**		6	45	

### Methodological quality assessment

**Fig. 6:**
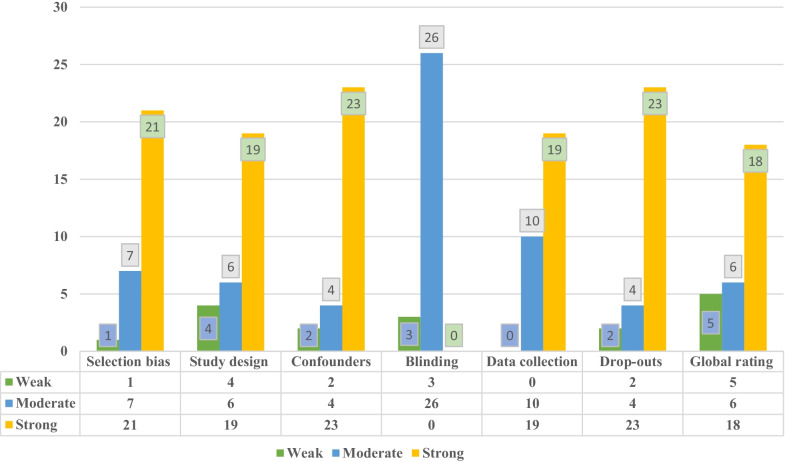
Risk of bias appraisal and quality assessment

The appraisal of qualities and risk of bias is shown in Fig. 6. According to the rating, 23 (79.31%) studies were strong in terms of cofounders and drop-out. Most studies (*n* = 19, 65.51%) were strong in terms of study design, and data collection (*n* = 19, 65.51%). Based on the global rating scores, 62.06% of the investigations were considered strong, 20.68% were considered moderate, and 17.24% were considered weak. Due to the nature of the interventions, which were educational approaches for patients after heart surgery, blinding of participants was not possible in most studies, but in some studies, blinding was performed as an evaluator.

## Discussion

Based on this review, educational approaches have the capacity and potential for self-monitoring and effective treatment of patients. In this systematic review, 29 papers (23 experimental and six observational-analytical) were reviewed in terms of the effects of educational interventions on patients with heart surgery.

These papers assessed a wide range of outcomes related to educational technologies, which were categorized into three main categories of patient-reported measures, clinical outcomes and economical outcomes. In general, most of the studies (28/29, 96.55%) had a significant impact on key outcomes such as improving the quality of care. In contrast, only one study did not report the intervention as effective.

Two of the most important consequences of educational platforms include reducing the level of anxiety and stress of patients after heart surgery and improving the care process [44]. Increasing the level of knowledge and awareness of patients after surgery such as heart surgery leads to changes in behavioral patterns, health and lifestyle [45]. Consequently, patient education is a structured, individual, and systematic process that assesses and transmits information to patients and their families that changes their health behavior and promote their well-being status [46]. The use of appropriate training technologies also reduces the cost of treatment and follow-up of individuals, and leads to a reduction in the workload of medical staff and care organizations [47]. Due to the progress of silent diseases, patient education has slowly become a significant concern, and hospitals and medical centers want to participate in the implementation of better education for patients and their families, and use the best emerging and advanced technologies for this purpose [9]. The use of educational approaches to educate patients after or before heart surgery has become common in recent years. Based on the results of selected studies, people who were trained both before and after heart surgery had significantly higher levels of preoperative knowledge than those who received training only after operation [48].

Studies by Kim et al. and Liu et al. distinguished the anxiety levels of patients who received and did not receive preoperative training; studies have revealed that patients who underwent preoperative training had lower levels of anxiety than those who did not. The patients in the intervention group cooperated more with health specialists and followed the procedures of the professionals [7, 49].

According to the studies, most of the solutions used to educate patients were based on video resources (as shown in Fig. 4). Educational videos do not require an actor or camera equipment, and it is relatively easy to add, remove or modify content in animated videos. The flexibility of videos to adapt clinical practices is a crucial variable [50]. Based on the studies reviewed in this study, video-based education can certainly support patient learning. However, more cumulative research is required to make evidence-based advances in the principles of video-based training in hospitals setting [51]. Based on the reviewed studies, virtual reality-based systems or environments have been used to educate patients. This technology causes patients to be immersed in the built environment, and facilities the learning of educational content in the best possible way [52]. Adopting a patient education system through the use of interactive technologies such as virtual and augmented reality in any organization is a significant and positive change that leads to improved quality of treatment and care [53].

There are strengths and limitations to this study. Strengths included using a search strategy with mesh terms that led to the identification of valuable studies. Also, the three authors extracted the data and screened the selected papers. This study also has two limitations. The first limitation is that non-English studies were excluded. Second limitation is that, the articles were retrieved based on a search of three databases, so some related studies may have been lost.

### Implications for practice

Educational approaches or technologies used to educate patients with various heart surgeries have replaced traditional approaches and teaching methods in recent years. However, due to the growing global need to use computer-based tools, this issue extends rapidly in modern countries. Therefore, it is suggested that developing countries should also provide a suitable platform for these studies. On the other hand, the cost of technology and innovative materials, as well as time, place and implementation methods vary greatly depending on the details of the application, but most of them are expensive and require enough space such as virtual environments or video resources. Furthermore, it is recommended that governments should plan for this type of training and related expenditures to improve patients’ knowledge or quality of life.

## Conclusion

In this systematic review, 29 articles related to the application of educational approaches and their effect on patients after heart surgery were analyzed. Almost all educational approaches and technologies have the functionality of reducing patient stress or anxiety and enhancing their satisfaction. Educational approaches have good potential to improve the quality of life and knowledge of patients. Therefore, technology and educational contents can be used as approaches to treatment management and care that aim to help control therapeutic interventions. Also, health policymakers are currently considering these technologies, because using them will reduce the financial burden on healthcare organizations.

## Data Availability

All data generated or analyzed during this study are included in this published article**.**
